# SMRT sequencing revealed to be an effective method for ADTKD-*MUC1* diagnosis through follow-up analysis of a Chinese family

**DOI:** 10.1038/s41598-020-65491-2

**Published:** 2020-05-25

**Authors:** Guo-qin Wang, Hong-liang Rui, Hong-rui Dong, Li-jun Sun, Min Yang, Yan-yan Wang, Nan Ye, Zhi-rui Zhao, Xue-jiao Liu, Xiao-yi Xu, Yi-pu Chen, Hong Cheng

**Affiliations:** Division of Nephrology, Beijing AnZhen Hospital, Capital Medical University, Beijing, 100029 China

**Keywords:** End-stage renal disease, Interstitial disease

## Abstract

We reported a large Chinese family diagnosed with autosomal dominant tubulointerstitial kidney disease caused by *MUC1* mutation (ADTKD-*MUC1*). Cytosine duplication within a string of 7 cytosines in the variable-number tandem repeats (VNTR) region of the *MUC1* gene was detected by long-read single-molecule real-time (SMRT) sequencing. MUC1 frameshift protein (MUC1fs) was found to be expressed in renal tubules and urinary exfoliated cells by pathological examination. The family, which consisted of 5 generations including 137 individuals, was followed for 5 years. Genetic testing was performed in thirty-four individuals, 17 of whom carried *MUC1* mutations. The ADTKD-*MUC1*-affected individuals had an elevated incidence of hyperuricaemia without gout attack. Within five years, higher baseline levels of urinary α1-microglobulin were detected in affected individuals with rapidly progressing renal failure than in affected individuals with stable renal function, and the increases manifested even before increases in serum creatinine. This study demonstrates that SMRT sequencing is an effective method for the identification of *MUC1* mutations. The pathological examination of MUC1fs expression in renal tissue and urinary exfoliated cells can contribute to early screening of family members suspected to be affected. It is suggested that affected individuals with elevated urinary α1-microglobulin levels should be closely monitored for renal function.

## Introduction

Autosomal dominant tubulointerstitial kidney disease (ADTKD) is a hereditary disease characterized by progressive tubulointerstitial nephropathy that ultimately leads to end-stage renal disease (ESRD)^1–4^. To date, four genes mutation which causes ADTKD have been identified: uromodulin (*UMOD*)^5^, mucin-1 (*MUC1*)^6^, renin (*REN*)^7^ and hepatocyte nuclear factor 1β (*HNF1B*)^8^. The disease forms caused by these mutations were named ADTKD-*UMOD*, ADTKD-*MUC1*, ADTKD-*REN* and ADTKD-*HNF1B*, respectively. Recently, a *SEC. 61A1* mutation was identified as a novel cause of ADTKD^9^.

The manifestations of ADTKD are nonspecific, including autosomal dominant inheritance, bland urinary sediment with absent or mild proteinuria, progressive kidney failure, and, in some cases, multiple small renal cysts^1–3,10,11^. Renal histology shows interstitial fibrosis and tubular atrophy, varying degree of nephrosclerosis and arteriolar thickening. Immunofluorescence or immunostaining on the renal tissue is negative. Electron microscopy usually showed irregular width and lamelation of the glomerular basement membrane and particular the tubular basement membrane^2,12–14^.

The diagnosis of ADTKD-*MUC1* depends on genetic analysis. The *MUC1* gene, located on chromosome 1q21, contains 20–125 copies of a repetitive sequence of 60 basepair in the variable-number tandem repeats (VNTR) domain^13^. Most *MUC1* mutations are caused by duplication of a cytosine within a seven-cytosine stretch in the VNTR region that produces a frameshift muatation, resulting in MUC1 frameshift protein (MUC1fs)^6,14–16^. In addition, mutations in other areas of the VNTR region or mutations outside the VNTR region have been reported^14,17^. Since the high guanosine/cytosine content and genomic architecture of the VNTR region, the MUC1 mutation is very difficult to analyze^6,10^. Recently, Wenzel *et al*. successfully located mutation sites related to ADTKD-*MUC1* using single-molecule real-time (SMRT) sequencing^18^. It is thought that the accumulation of MUC1fs in renal tubular epithelial cells may be an important pathogenic mechanism of ADTKD-*MUC1*^2,18^. Notably, positive MUC-1fs staining in renal tubular epithelial cells and urinary exfoliated cells may be helpful for the diagnosis of ADTKD-*MUC1*^14^.

Among the four types of ADTKD, ADTKD-*UMOD* patients frequently present hyperuricaemia with frequent gout. ADTKD-*REN* is characterized by childhood anaemia. Patients with *HNF1B* mutations develop multiple extrarenal manifestations. Thus far, ADTKD-*MUC1* has not been found to exhibit specific clinical features, and heterogeneity can be found among different families or races^4^. There have been no published reports about the clinical manifestations in Chinese families with ADTKD-*MUC1*. Here, we reported a family of the Chinese Han population with ADTKD. We identified a cytosine duplication mutation of the VNTR region in the *MUC1* gene by long-read SMRT sequencing and confirmed the expression of MUC1fs in renal biopsy tissue and even in urinary exfoliated cells. We followed this Chinese family for 5 years to investigate the clinical characteristics of ADTKD-*MUC1*.

## Methods

### Family survey

Family surveys were conducted through personal interviews with family members. The clinical information of deceased affected individuals was obtained from their family members. The clinical histories, blood and urine laboratory test results and imaging data of 37 individuals of the family were collected many times over a 5-year period. Thirty-four individuals in the family accepted genetic testing. Two patients, including the proband (IV-6) and her son (V-6), underwent renal biopsy and pathological examination. The diagnostic criteria of ADTKD for the proband and her son were those proposed by the KDIGO^2^ and included renal histology findings, clinical manifestations and a family history of autosomal dominant inheritance. Twenty-eight individuals in the family were followed for 5 years. Laboratory tests of blood and urine were repeated in the fifth year.

The study was approved by the Ethics Review Committee of Beijing Anzhen Hospital, Capital Medical University (protocol no. 2015009×), and implemented in accordance with the Declaration of Helsinki. The individuals enrolled in this study all gave written informed consent.

### PCR amplification of the VNTR region

Fragments of the *MUC1* VNTR region were directly PCR-amplified from genomic DNA by using two primers (PS2: 5′-GGAGAAAAGGAGACTTCGGCTAC CCAG-3′ and PS3: 5′-GCCGTTGTGCACCAGAGTAGAAGCTGA-3′). Briefly, 50 ng of DNA template was mixed with 25 µl of T8 2× High Fidelity Master Mix (Tsingke Biotech, Beijing), 10 µM primers, 10 µl of 5× High GC buffer (Tsingke Biotech) and ddH_2_O to a total volume of 50 µl. Then, the samples were amplified via initial denaturation at 98 °C for 5 min; 30 cycles of amplification at 98 °C for 10 s and 74 °C for 4 min (5 cycles)/72 °C for 4 min (5 cycles)/70 °C for 4 min (5 cycles)/68 °C for 4 min (15 cycles); and a final extension at 68 °C for 10 min before being held at 4 °C. The PCR products were subjected to gel electrophoresis, and DNA bands over 2800 bp in size were excised. The DNA was extracted and purified with a DNA Gel Extraction Kit (CWBio, Beijing) for sequencing.

### SMRT sequencing of the proband VNTR region

SMRTbell libraries were constructed by using the PCR products of the *MUC1* VNTR region and a SMRTbell^TM^ Template Prep Kit 1.0 (Pacific Biosciences, CA) according to the manufacturer’s instructions. Three-microgram samples of the purified PCR products of the VNTR region were treated according to the Pacific Biosciences template preparation and sequencing protocol. A Binding Kit 2.1 V2 (Pacific Biosciences) and v3 primers were used to make SMRTbell templates bound to polymerases. The polymerase-template complexes were loaded by the diffusion loading method, and SMRT sequencing was then carried out on a Sequel® real-time sequencer by using a Sequencing Kit 2.1 V2 (Pacific Biosciences). All movie lengths were set to 1200 min for each SMRT cell. Bioinformatics analysis of SMRT sequencing was completed by Beijing GrandOmics BioSciences Co.

### Sanger sequencing

To confirm the *MUC1* VNTR mutations in other family members, Sanger sequencing was performed. For Sanger sequencing, the PCR products of the VNTR region were amplified with intronic primers (5’-ACAGGATGTCACTCTGGC-3’) and sequenced using Applera BigDye version 3.1 (Applied Biosystems, CA) with 5× high-GC buffer (Tsingke Biotech) and an ABI 3730XL Avant Genetic Analyzer automated sequencer (Thermo Fisher Scientific). Two researchers analysed the sequences independently. For individuals with *MUC1* VNTR mutations and normal renal function (as assessed by estimated glomerular filtration rate (eGFR) measurement), we re-collected blood samples and performed Sanger sequencing again at a different company.

### Whole-exome sequencing

Genomic DNA was prepared from peripheral leukocytes of three affected individuals (IV-6, IV-34 and V-6) and two unaffected individuals (IV-8 and IV-36). Further details are provided in the Supplementary Methods.

### Generation of MUC1fs antibodies

A rabbit anti-human MUC1fs polyclonal antibody was commercially prepared by Beijing Protein Innovation. A peptide antigen corresponding to 20 residues (NH2-SPRCHLGPGHQAGPGLHRPP-OH) of MUC1fs^14,16^ was chemically synthesized and used to immunize rabbits for antibody production. Next, the rabbits were immunized with peptide-conjugated bovine serum albumin (BSA) by multiple intradermal injections. The rabbit serum was applied to a peptide-conjugated column. Bound IgG was eluted with 0.1 M glycine-HCl (pH 2.5) and immediately adjusted to pH 8.0. The antibody titres were determined by enzyme-linked immunosorbent assay using the peptide antigen as the solid phase.

### Immunohistochemical staining of renal tissue

Paraffin-embedded kidney tissues were cut into 4 μm-thick sections and then processed by autoclave heating-induced epitope retrieval (in citrate solution, pH 6.0). A rabbit anti-human MUC1 polyclonal antibody (1:400, Zsbio, Beijing) or a rabbit anti-human MUC1fs polyclonal antibody (1:1000) was used as the primary antibody, and a horseradish peroxidase (HRP)-labelled goat anti-rabbit IgG antibody (1:400, Zsbio) was used as the secondary antibody. Nuclei were stained with haematoxylin. Renal biopsy tissues of the other three cases with thin glomerular basement membrane nephropathy were stained as controls.

### Immunofluorescence staining of renal tissue

For double staining for an indirect immunofluorescence assay of the renal tubular epithelial cell marker protein Cytokeratin-19 (CK19) and other proteins, frozen renal biopsy tissues from V-6 were fixed in 4% paraformaldehyde, cut into 5 μm-thick sections, permeabilized with 0.1% Triton X-100 and blocked with 2% BSA. After blocking, the sections were incubated overnight at 4 °C with rabbit monoclonal anti-MUC1 (1:400) or anti-MUC1fs (1:1000) and mouse polyclonal anti-CK19 (1:400, Zsbio) antibodies and then washed with PBS three times. Next, the sections were incubated with rhodamine-labelled goat anti-rabbit (ZSbio) and FITC-labelled sheep anti-mouse (Zsbio) secondary antibodies for 2 h at room temperature. After staining, the tissue sections were observed with a fluorescence microscope (Nikon, Japan).

### Immunostaining of urinary exfoliated cells

The immunostaining staining procedure was performed according to the methods of Živná, M. *et al*.^14^. Briefly, 150 ml samples of second morning urine were chilled at 4 °C for 1 h and centrifuged at 3500 RPM for 10 min, and then the supernatant was removed. The pellets were washed with 5 ml of wash buffer (2 mM EDTA and 0.1% BSA in PBS) and centrifuged again at 3500 RPM for 10 min. The supernatant was removed, and the pellets were resuspended in 150 μl of wash buffer. Thirty microliters of each suspension was transferred to a Polysine adhesive microscope slide (Thermo Fisher Scientific). The slides were dried at room temperature for 30 min, fixed with ice-cold 100% methanol for 10 min, and blocked with 5% FBS with 0.05% Tween 20 in PBS for 45 min. After protein blocking, the slides were incubated with a rabbit monoclonal anti-MUC1 (1:400) or anti-MUC1fs (1:1000) antibody and a mouse polyclonal anti-CK19 (1:400, Zsbio) antibody and then washed with PBS three times. Next, the sections were incubated with rhodamine-labelled goat anti-rabbit (Zsbio) and FITC-labelled sheep anti-mouse (Zsbio) secondary antibodies for 2 h at room temperature. After staining, the tissue sections were observed with a fluorescence microscope (Nikon, Japan).

### Statistical analysis

Continuous variables with normal distributions are expressed as the means ± standard deviations (SDs). For categorical data, the frequency and percentage are reported. Quantitative variables were compared using t-tests. Variables with non-normal distributions are presented as the medians and interquartile ranges, and Wilcoxon-Mann-Whitney tests were used for univariate comparison. Statistical analysis was performed using SPSS 21.0 statistical software. *P* < 0.05 was considered to indicate statistical significance.

## Results

### Clinical and renal histopathological data of the proband and her son

**Figure 1:**
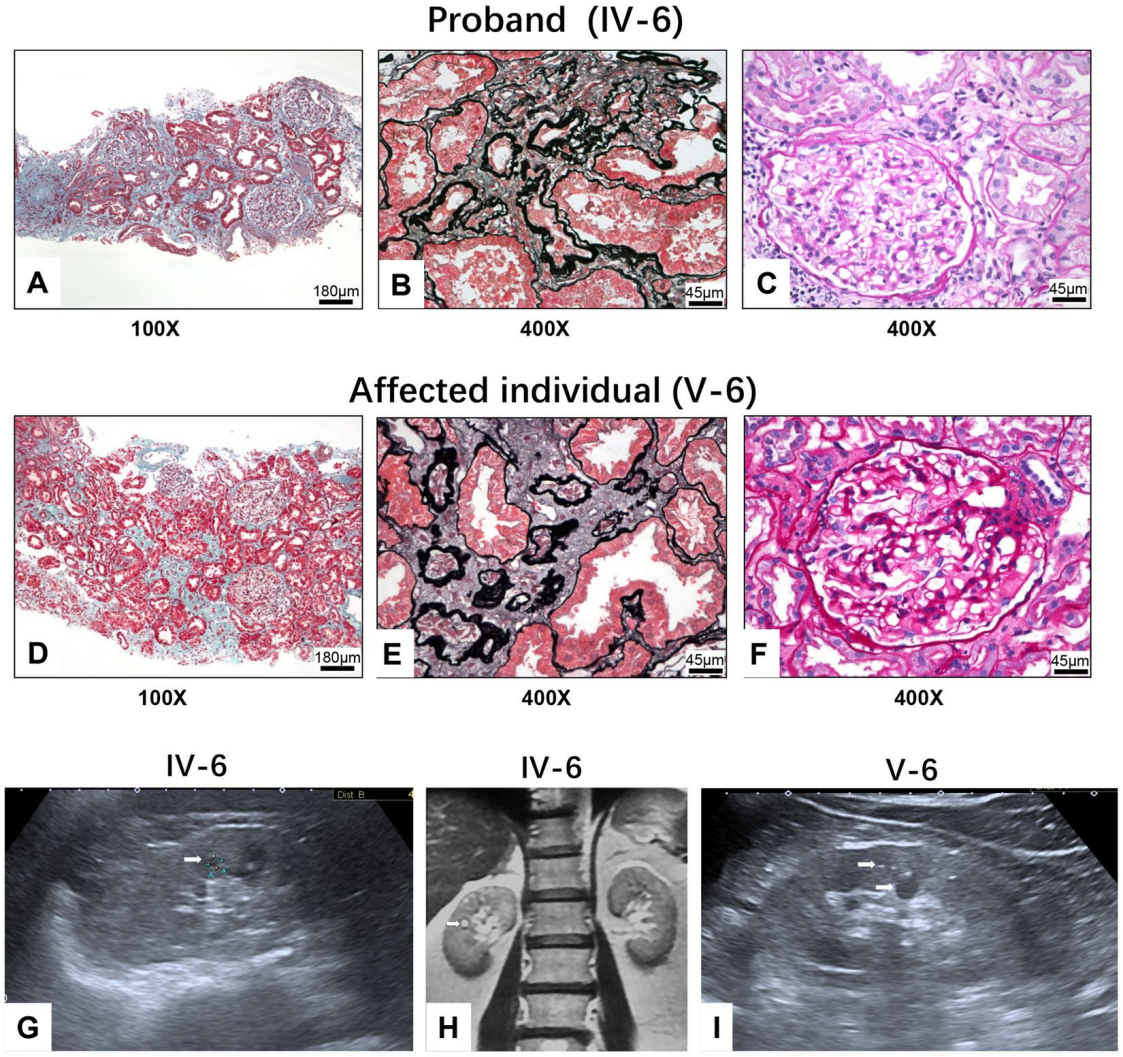
Renal pathological manifestation and image finding. (**A**) to (**F**), Results of renal biopsy pathological examinations of the proband (IV-6) (**A** to **C**) and her son (V-6) (**D** to **F**). (**A**) and (**D**), Extensive interstitial fibrosis (Masson trichrome stain 100×, Bar = 180 μm); (**B**) and (**E**), Tubular atrophy and tubular basement membrane thicken (periodic acid-silver methenamine stain 400×, Bar = 45 μm); (**C**) and (**F**), Normal glomerulus (periodic acid Schiff stain 400×, Bar = 45 μm). (**G**) to (**I**), Renal ultrasound showed small cysts could be detected in kidney (white arrow) of IV-6 (**G**) and V-6 (**I**); renal magnetic resonance imaging (MRI) shows small cysts and calcification in IV-6 (H) (white arrow).

The proband (IV-6) was a 55-year-old female who had exhibited hypertension and mildly elevated serum creatinine (SCr) levels for 7 years. Physical examination revealed no significant abnormal findings. Her urinalysis showed a protein level of 0.2 g/d without red blood cells. The level of SCr was 161 μmol/L, the eGFR was 31.1 ml/min/1.73 m^2^ according to the Chronic Kidney Disease Epidemiology Collaboration (CKD-EPI) equation, and the serum uric acid level was 475 μmol/L. The haemoglobin concentration was 117 g/L. The serum antinuclear antibody and antineutrophil cytoplasmic antibody were negative. Ultrasonography and MRI of the kidneys revealed multiple small cysts and normal kidney length (Fig. 1G,H). Pathological examination of renal biopsy tissue showed extensive interstitial fibrosis and tubular atrophy with thickening of the tubular basement membrane (Fig. 1A,B). The glomeruli were normal (Fig. 1C) or exhibited ischaemic sclerosis. No positive findings were observed on immunofluorescence staining for immunoglobulins, complement C3 and C1q. Electron microscopy also displayed obvious tubulointerstitial fibrosis.

In the same year, her 29-year-old son (V-6) was admitted to the hospital for abnormal renal function. Laboratory tests showed that his haemoglobin concentration was 144 g/L, his SCr concentration was 121 μmol/L, his eGFR was 69.3 ml/min/1.73 m2 according to the EPI equation, and his uric acid concentration was 545.8 μmol/L. Urinalysis showed bland urine with a random urine microalbumin-creatinine ratio of 6.05 mg/g. Renal biopsy was performed. The immunofluorescence examination results were negative. The glomeruli were normal or exhibited ischaemic sclerosis. Cord-like renal interstitial fibrosis and tubular atrophy were observed and were accompanied by small focal mononuclear cell infiltration and mild arteriole hypertrophy (Fig. 1D–F). He was diagnosed with chronic tubulointerstitial nephropathy, as same as his mother. In addition, V-6 exhibited bilateral multiple renal cysts on ultrasonography, mainly in the medulla and corticomedullary area, and calcification of the cystic wall (Fig. 1I).

### Family surveys showed that many individuals in the family suffered from renal insufficiency, dialysis or death from uraemia

**Figure 2:**
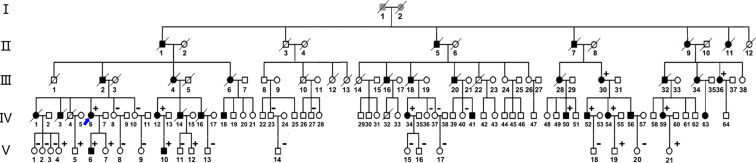
Family pedigree. Square symbols and circle symbols indicate males and females respectively. Black symbols indicate affected members, white symbols indicated unaffected members and slashed symbols represent deceased individuals. The individual pointed by blue arrow is proband. The results of *MUC1* gene sequencing were marked with black in the upper right, + means with mutation of *MUC1*, − means without mutation of *MUC1*. The information of death with renal failure in first and second-generation individuals was obtained by visiting their posterity.

By inquiry of family history, we learned that the proband’s father, one brother and one sister were died of ESRD several years ago. Next, we conducted a family survey. This family had five generations including one hundred and thirty-seven individuals, of which thirty-six individuals had died (eighteen died of renal insufficiency). We obtained clinical information on the deceased affected individuals from their descendants. Fourteen living individuals (8 females and 6 males) with ages ranging from 29 to 75 years (median: 46 years) were initially known to have renal insufficiency or to have undergone maintenance haemodialysis. A total of thirty-seven living individuals (19 females and 18 males) received blood and urine testing, and their ages ranged from 20 to 75 years. Twelve of them (7 females and 5 males) had renal insufficiency or had undergone maintenance haemodialysis (Fig. 2). Genetic testing was performed on thirty-four individuals.

### SMRT sequencing identified a cytosine insertion in MUC1-VNTR in the proband and her son

**Figure 3:**
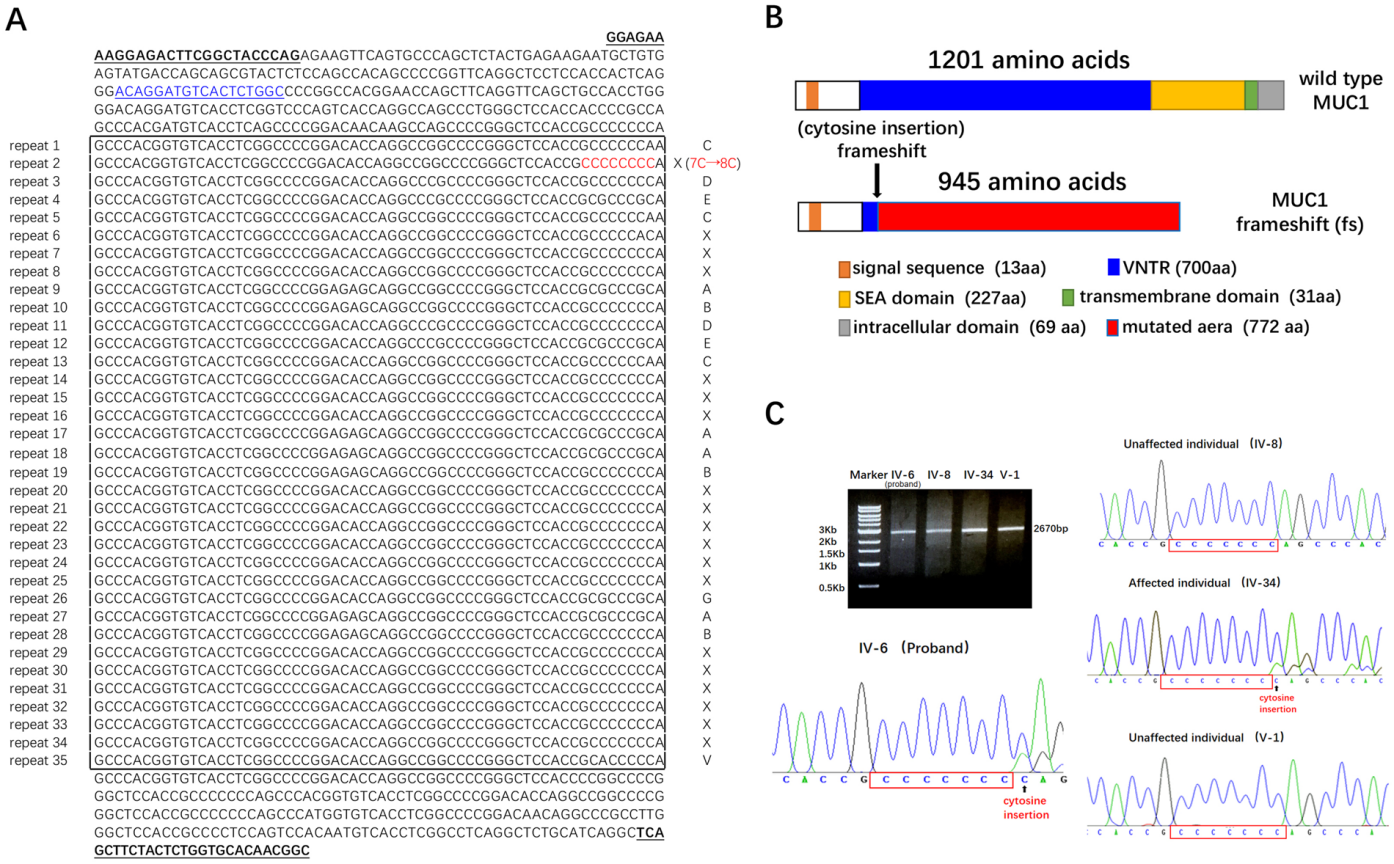
The sequencing results of *MUC1* VNTR region by SMRT sequencing and direct Sanger sequencing. (**A**) The complete VNTR region sequence of the proband (IV-6). The inserted cytosine (7 C → 8 C) was detected in the second repeat consensus (highlighted in red). Capital letter code of the 60 mer VNTR-units according to Kirby *et al*.^6^ is displayed next to each repeat sequence. Underlined and bolded sequences are the locations of the amplification primers PS2 and PS3; underlined and blue sequences are the location of Sanger sequencing primer. (**B**) Scheme of the wild-type and mutated *MUC1* allele products as occurred in the affected individuals (dark blue boxes represent the wild-type VNTR domain, and red boxes represent the frameshift motif of the mutated *MUC1*). The arrow indicates the distinct VNTR repeat where the cytosine insertion (+C) occurred. (**C**) Long range PCR amplification of the *MUC1*-VNTR fragments with IV-6, IV-8, IV-34 and V-1 were separated by agarose gel electrophoresis (1.0% gel). The size of PCR product is about 2670 bp as indicated. Wild type *MUC1* VNTR without cytosine insertion was identified with IV-8 and V-1; the insertion of cytosine (7 C → 8 C, black arrow) was identified in IV-6 and IV-34. The red box indicates the normal string of seven cytosines.

Blood samples were obtained from the proband and her son for genetic testing. Mutations in *UMOD, REN, HNF1B* and *SEC. 61A1* were not found by whole-exome sequencing (see Supplementary Data). Therefore, SMRT sequencing technology was used for assessment of cytosine insertions in the VNTR region of *MUC1*. The SMRT sequencing results showed that the VNTR region of the proband contained 35 repetitions of 60 nucleic acids (Fig. 3A). A cytosine insertion that caused a 7C-to-8C mutation occurred in the second repeat, which resulted in a frameshift in the VNTR region and the appearance of a newly encoded protein, MUC1fs. Compared with wild-type *MUC1*, the mutated *MUC1* lacked C-terminal domains, which may have led to a functional change in MUC1 (Fig. 3B).

All exons of *MUC1* were also examined by direct Sanger sequencing, and no other mutations were found.

### Sanger sequencing for verifying the mutation of the MUC1 VNTR region in family members

Since we found that the location of the VNTR mutation site was approximately 400 bp from the start site of PCR amplification, which is within the limits of direct Sanger sequencing, we used Sanger sequencing to verify the VNTR mutation in 34 of 37 family members (including the proband and her son) (Fig. 3C). Eighteen individuals (18/34) had insertion of one cytosine at the same location in the VNTR region of the *MUC1* gene. Aside from the 34 individuals, the other three individuals (IV-28, IV-29 and IV-56) did not undergo genetic testing. IV-28 and IV-29 had normal renal function and were presumed to be unaffected given that none of their immediate family members had abnormal renal function. We speculated that IV-56 was an affected individual since he had elevated SCr, his father had renal insufficiency and his brother had an *MUC1* mutation that was confirmed by genetic testing. Overall, among the thirty-seven individuals, eighteen (9 females and 9 males) were unaffected. Nineteen (10 females and 9 males) were affected, including seven (3 females and 4 males) without clinical and laboratory manifestations.

### Detection of MUC1fs in renal biopsy tissue and urinary exfoliated cells of affected individuals

**Figure 4:**
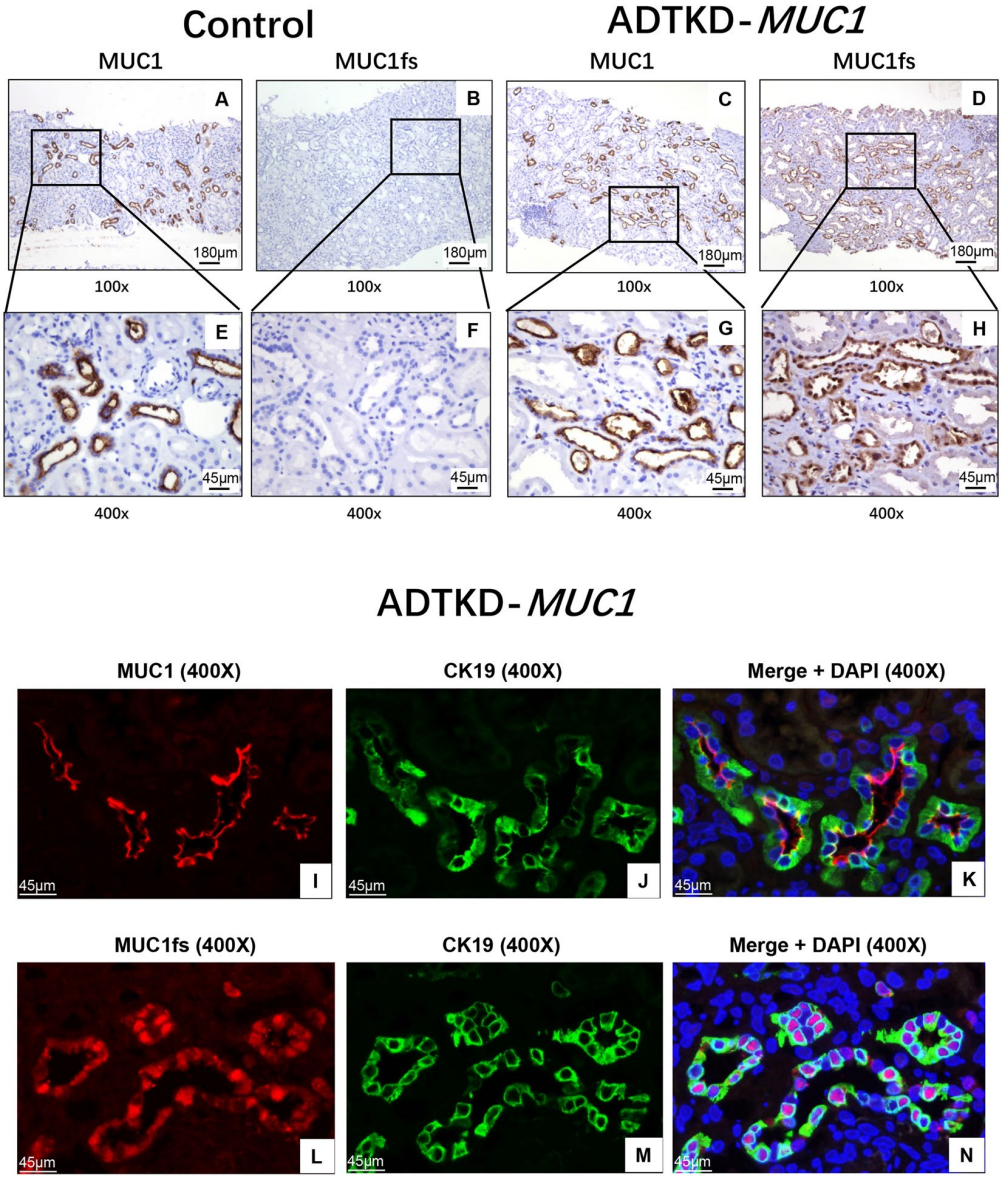
Expression of MUC1fs with renal biopsy tissue. (**A**) and (**C**), Immunohistochemically staining showing the positive staining of wild type mucin-1 in renal tubules with control (**A**) and proband (**C**) (100×, Bar = 180 μm); (**E**) and (**G**), Magnified view showing renal tubules positive staining of MUC1 with control (**E**) and proband (**G**) (400×, Bar = 45 μm). (**B**) and (**D**), Immunohistochemically staining of MUC1fs in renal tubules of negative staining with control (**B**) and proband (**D**) (100×, Bar = 180 μm); (**F**) and (**H**), Magnified view showing renal tubules positive staining of MUC1fs of negative staining with control (**F**) and proband (**H**) (400×, Bar = 45 μm). (**I**) to (**N**), double immunofluorescence staining of Cytokeratin 19 (CK19) with MUC1 or MUC1fs in tubular of V-6. The localization of CK19 (green spots), MUC1 or MUC1fs (red spots) and merged image with DAPI (blue spots) with frozen renal sections were shown as indicated (400×, Bar = 45 μm).

Immunohistochemical staining of wild-type MUC1 and MUC1fs was carried out on renal biopsy tissues of the proband (IV-6) and her son (V-6). We found positive staining for wild-type MUC1 and MUC1fs in the renal tubules of IV-6 and V-6. Three patients with thin basement membrane nephropathy were selected as the control patients. Wild-type MUC1 staining was positive in the renal biopsies of the control patients, while MUC1fs staining was negative (Fig. 4A–H). In addition, double immunofluorescence staining of MUC1 and CK19, a marker of epithelial cells^19^, showed that wild-type MUC1 localized along the luminal surfaces of renal tubular epithelial cells. In contrast, we found that MUC1fs was expressed in both the cytoplasm and nuclei of renal tubular epithelial cells, unlike wild-type MUC1 (Fig. 4I–N).

**Figure 5:**
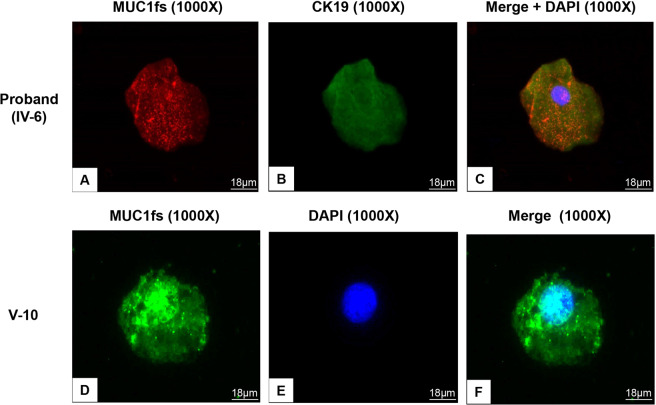
Immunofluorescence staining of MUC1fs with urinary exfoliated cells from ADTKD-*MUC1* individuals. (**A**) to (**C**), Double immunofluorescence staining in urinary exfoliated cells of the proband (IV-6) with MUC1fs (red spots), CK19 (green) and merge+DAPI (1000×, Bar = 18 μm); (**D**) to (**F**), Immunofluorescence staining in urinary exfoliated cells of the affected individual (V-10) with MUC1fs (green spots), DAPI(blue) and merge (1000×, Bar = 18 μm).

We further performed immunofluorescence staining of urinary smears of the proband and found expression of MUC1fs in urinary exfoliated cells. CK19 staining in these cells was also positive, indicating that they were tubular epithelial cells (Fig. 5A–C). Subsequently, the urinary exfoliated cells of another affected individual (V-10) were analysed, and MUC1fs staining was also positive (Fig. 5D–F).

### Age at onset of renal insufficiency and progression to ESRD in affected individuals

Among the nineteen affected individuals with *MUC1* mutations, twelve (7 females and 5 males) had abnormal renal function, and the age at onset of renal insufficiency ranged from 29 to 75 years (median: 46 years). Aside from V-6, whose age at onset was 29 years, all of the other affected individuals were over 35 years old at onset. The ages of affected individuals who progressed to ESRD ranged from 45 to 59 years (median: 52.5 years) (Table 1). Seven affected individuals with normal renal function were also under the age of 35. Eighteen unaffected individuals all had normal renal function, with ages ranging from 25 to 56 years (median: 42 years).

**Table 1:** Laboratory examinations of members with *MUC1* mutation for 5-year follow-up.

Family member	Baseline Age (1^st^ year)	Sex	Age at diagnosis (years)	Age at onset of ESRD (years)	Serum creatinine (μmol/L)		eGFR (CKD-EPI) (ml/min·1.73 m^2^)		Serum uric acid (μmol/L)		Urine α1-microglobulin (mg/L)	
					1^st^ year	5^th^ year	1^st^ year	5^th^ year	1^st^ year	5^th^ year	1^st^ year	5^th^ year
III-30	75	F	75	—	156.5	174.4	27.6	23.4	327.8	367.6	25.0	19.5
III-36	70	F	52	59	*dialysis*							
IV-6	53	F	52	—	161.0	294.7	31.1	15.5	475.5	433.1	13.5	68.1
IV-12	58	F	54	—	102.0	247.1	52.8	17.3	435.2	499.5	50.4	111.0
IV-34	53	F	45	51	*dialysis*							
IV-41	46	M	46	48	611.0	*dialysis*	8.7	—	558.0	—	98.9	—
IV-50	50	M	50	53	180.0	*dialysis*	37.0	—	502.2	—	82.1	—
IV-52	51	M	51	—	121.8	131.3	58.9	51.9	432.8	456.1	17.2	67.9
IV-54	48	F	42	45	*dialysis*			—				
IV-56	45	M	45	—	130.2	N/A	54.7	N/A	473.8	N/A	21.1	N/A
IV-59	45	F	44	49	183.0	*dialysis*	27.4	—	469.5	—	47.9	—
V-6	29	M	29	—	121.0	145.4	69.3	53.6	545.8	438.4	69.6	81.4
V-4	31	F	—	—	55.6	73.4	119.6	90.7	399.4	364.3	26.1	25.4
V-5	28	M	—	—	66.7	71.5	124.5	116.8	426.7	425.0	11.5	21.7
V-7	27	F	—	—	60.6	60.3	118.7	115.6	377.5	542.9	22.5	5.3
V-10	32	M	35	—	86.0	292.5	102.5	22.5	466.7	341.8	146.7	169.0
V-12	26	M	—	—	107.2	84.6	81.9	105.3	501.4	643.9	102.0	71.1
V-19	25	M	—	—	64.0	N/A	129.4	N/A	381.8	N/A	38.8	N/A
V-21	25	F	—	—	59.2	62.1	122.1	115.8	321.4	309.5	18.1	9.9

Normal range: serum creatinine 41.0–81.0 μmol/L for female, 57.0–111.0 μmol/L for male; serum uric acid 155.0–357.0 μmol/L for female, 208.0–420.0 μmol/L for male; urine α1-microglobulin 0–20 mg/L. Individuals below the dotted line are the mutant MUC1 carriers with normal renal function at 1^th^ year. IV-56 and V-19 did not participate in follow-up for personal reasons.

### Comparison of baseline serum uric acid levels in affected individuals and unaffected individuals

**Figure 6:**
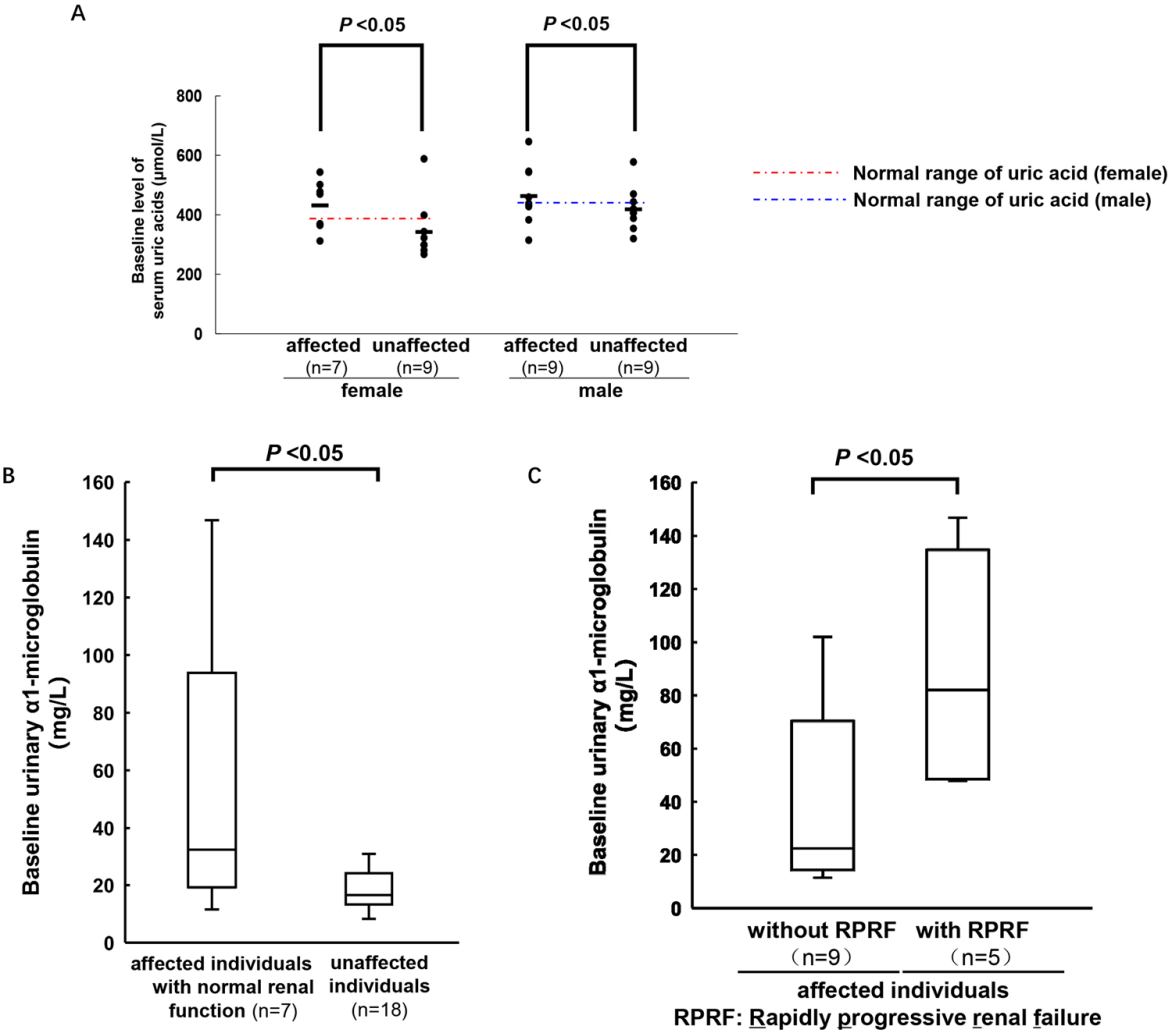
Comparison of the level of serum uric acid and urinary α1-microglobulin in this family. (**A**) The family members were divided into four groups based on gender and gene mutation affected or not: affected female (n = 7), unaffected female (n = 9), affected male (n = 9) and unaffected male (n = 9). The average baseline levels of serum uric acid were compared as indicated. (**B**) The median baseline levels of urinary α1-microglobulin were compared in unaffected individuals (n = 18) and affected individuals with normal renal function (n = 7). (**C**) Affected individuals were divided into two groups: with RPRF (n = 5) and without RPRF (n = 9). The median baseline levels of urinary α1-microglobulin were compared. RPRF, Rapidly progressive renal failure, which defined as double serum creatinine or entering ESRD within 5 years.

The incidence of hyperuricaemia was 84.2% (16/19) in affected individuals of this family and even reached 71.4% (5/7) in affected individuals with normal renal function. The average uric acid level of affected individuals (except dialysis individuals) was 456.1 ± 63.5 μmol/L. However, no gout attack happened among these individuals. The incidence of hyperuricaemia was only 22.2% (4/18) in unaffected individuals, and the average serum uric acid level was 342.8 ± 103.8 μmol/L, which was significantly different from that of affected individuals (*P* < 0.05). The uric acid level of affected male individuals was higher than that of unaffected male individuals (464.27 ± 98.6 μmol/L vs 419.1 ± 67.3 μmol/L, *P* < 0.05), and the results of comparison between affected individuals and unaffected individuals in females (432.7 ± 85.5 μmol/L vs 342.7 ± 59.6 μmol/L, *P* < 0.05) were consistent with those in males (Fig. 6A). However, the incidence of hyperuricaemia and the levels of uric acid in affected male individuals were similar to those in affected female individuals (88.9% vs 90%, *P* > 0.05, and 464.27 ± 98.6 μmol/L vs 432.7 ± 85.5 μmol/L, *P* > 0.05).

### Urinary α1-microglobulin might be an early indicator of ADTKD-MUC1

All individuals with *MUC1* mutations (except those undergoing dialysis) had no microscopic haematuria and little or no proteinuria. An elevated level of α1-microglobulin in urine, a marker of proximal renal tubular damage, was found in 75.0% (12/16, excluding individuals undergoing dialysis) of affected individuals and was even found in 71.4% (5/7) of affected individuals with normal renal function (Table 1). The above results are consistent with the characteristics of chronic tubulointerstitial disease. In addition, the urine tests were normal for all unaffected individuals.

Interestingly, compared with unaffected individuals, affected individuals with normal renal function had similar eGFRs (108.5 ± 22.0 ml/min/1.73m^2^ vs 115.8 ± 10.9 ml/min/1.73m^2^, *P* > 0.05) but significantly elevated urinary α1-microglobulin levels (32.5 mg/L (19.2, 93.9) vs 16.5 mg/L (13.3, 24.2), *P* < 0.05) (Fig. 6B). In addition, compared with affected individuals with stable renal function during the 5-year follow-up, affected individuals with SCr doubling or progression to ESRD within follow-up had higher baseline levels of urinary α1-microglobulin (23.8 mg/L (11.5, 102.0) vs 82.1 mg/L (47.9, 146.7), *P* < 0.05) (Fig. 6C).

### Progression of renal dysfunction in affected individuals during 5-year follow-up

Twenty-eight family members who completed genetic testing, including seventeen affected individuals and eleven unaffected individuals, were followed for five years. With regard to the baseline clinical manifestations of the seventeen affected individuals, three of them (3/17) were already on haemodialysis, seven (7/17) had renal insufficiency, and seven (7/17) had normal renal function. An unaffected individual (III-3) died of pneumonia during the five-year follow-up.

After five years, the eGFR of thirteen affected individuals without dialysis (13/14) had decreased by 20.73 ± 22.65 ml/min/1.73 m^2^; this decrease was significantly greater in magnitude than that for unaffected individuals (6.42 ± 3.89 ml/min/1.73 m^2^). Three affected individuals (3/17) with renal insufficiency progressed to ESRD that required dialysis. Among seven affected individuals with normal renal function, one male individual (V-10) rapidly progressed to stage 4 CKD in the 4^th^ year of follow-up, but the others retained normal renal function (Table 1). In addition, a 31-year-old affected individual (V-12) had increased serum uric acid and urinary α1-microglobulin levels, while his eGFR remained steady.

## Discussion

ADTKD-*MUC1* is a rare genetic disorder. The diagnosis of ADTKD-*MUC1* is limited mostly due to the difficulty of MUC1 genetic testing; thus, the specific clinical manifestations lack characterization, especially in Chinese families. The families with ADTKD-*MUC1* reported thus far have predominantly been of European or European-American origin, whereas others have been of Australian, African American, Middle Eastern, Israeli, Hispanic, Italian, and Native American origin^13,20–22^. In this study, we investigated and followed a large Chinese Han family with ADTKD for 5 years and identified a *MUC1* mutation by SMRT sequencing.

Because of complex structure of the VNTR region in the *MUC1* gene, mutations in the *MUC1* gene has been impossible to analyse by straight Sanger sequencing, whole-genome, or whole-exome massive parallel sequencing^10^. In this study, we identified a cytosine duplication within one of the VNTR sequences by SMRT sequencing combined with Sanger sequencing, which confirmed the existence of the *MUC1* mutation. Subsequently, immunohistochemical staining of MUC1fs was found to be positive in renal tubular epithelial cells, which provided the further evidence of the *MUC1* mutation^22,23^. Due to difficulties in sequencing the VNTR region of *MUC1*and the risk of renal biopsy in suspected patients with renal insufficiency, Živná M *et al*.^14^ developed an immunostaining method for MUC1fs in urinary exfoliated cells for screening of ADTKD-*MUC1*. In this study, the proband and an affected individual’s urinary exfoliated cells were determined to be MUC1fs positive by this method, which supported the effectiveness of this non-invasive testing method for screening suspected individuals.

We followed up with family members for 5 years to analyse their clinical characteristics. The age at onset of renal insufficiency with ADTKD-*MUC1* reported by Kiser ranged from 34 to 65 years^24^. In our family, all affected individuals with renal insufficiency except V-6 were over 35 years of age. During the five-year follow-up, only V-10 developed renal insufficiency at over 35 years of age; the other affected individuals under 35 years of age retained normal renal function. This suggests that renal function in affected individuals should be monitored more frequently after 35 years of age than before 35 years of age.

In previous studies, the age at onset of ESRD has been variable in individuals with ADTKD-*MUC1*^3,10–13,21,24^. In our study, six affected individuals (2 females and 4 males) progressed to ESRD or began dialysis at age ranging from 45 to 59 years old. Three highly suspected family members without genetic testing also started dialysis within this age range. During the follow-up, two affected individuals progressed to ESRD at the age of 53 years. Overall, the age at onset of ESRD in this family was mainly between 45 and 59 years, with a median age of 53.5 years, unlike variable age at onset of ESRD with families reported previously^13^.

The rate of decline in renal function in affected individuals varied greatly. The two individuals (IV-12 and V-10) with the fastest decreases in eGFR were a mother and child. This mother-son pair lived in the same area as other family members, and their habits were similar. It is thus speculated that some other unknown genetic or environmental factors may affect disease progression^22^.

Unlike that in ADTKD-*UMOD*, hyperuricaemia in ADTKD-*MUC1* has no specificity^2^. In this family, the incidence of hyperuricaemia in affected individuals, even those with normal renal function, was significantly higher than that in unaffected individuals, and there was no difference between males and females. Although gout attack has been previously reported in ADTKD-*MUC1* patients^12,24^, none of the individuals in this family had gout attacks. Although hyperuricaemia is not a characteristic manifestation of ADTKD-*MUC1*^2^, the high incidence of hyperuricaemia in this family suggests that its pathophysiologic significance should be emphasized.

To date, the clinical characteristics of ADTKD-*MUC1*, unlike those of other types of ADTKD, have still not been summarized^4^. Interestingly, we found that affected individuals with normal renal function also had increased levels of urinary α1-microglobulin. More importantly, within the five-year follow-up, we found that the affected individuals with rapidly progressing renal failure, who all had higher baseline urinary α1-microglobulin levels than the affected individuals with stable renal function. The elevation in urinary α1-microglobulin has not been reported in previous studies, suggesting that urinary α1-microglobulin may be not only an early indicator of renal insufficiency but also a predictor of the progression of renal dysfunction. The level of urinary α1-microglobulin reflects the function of the proximal tubule and has also been shown to be elevated in other tubulointerstitial nephropathies^25^. Therefore, it is necessary to validate its value in additional families with ADTKD-*MUC1*. At the least, these findings suggest that renal function in affected family members with elevated urinary α1-microglobulin levels should be closely monitored.

Many researchers have pointed out that kidney cysts are not characteristic of ADTKD-*MUC1*^11,12,26^. In this family, renal cysts and renal cyst wall calcification were detected by image examination in some affected individuals. However, further follow-up data should be collected and additional family studies should be performed to assess this clinical feature.

**Figure 7:**
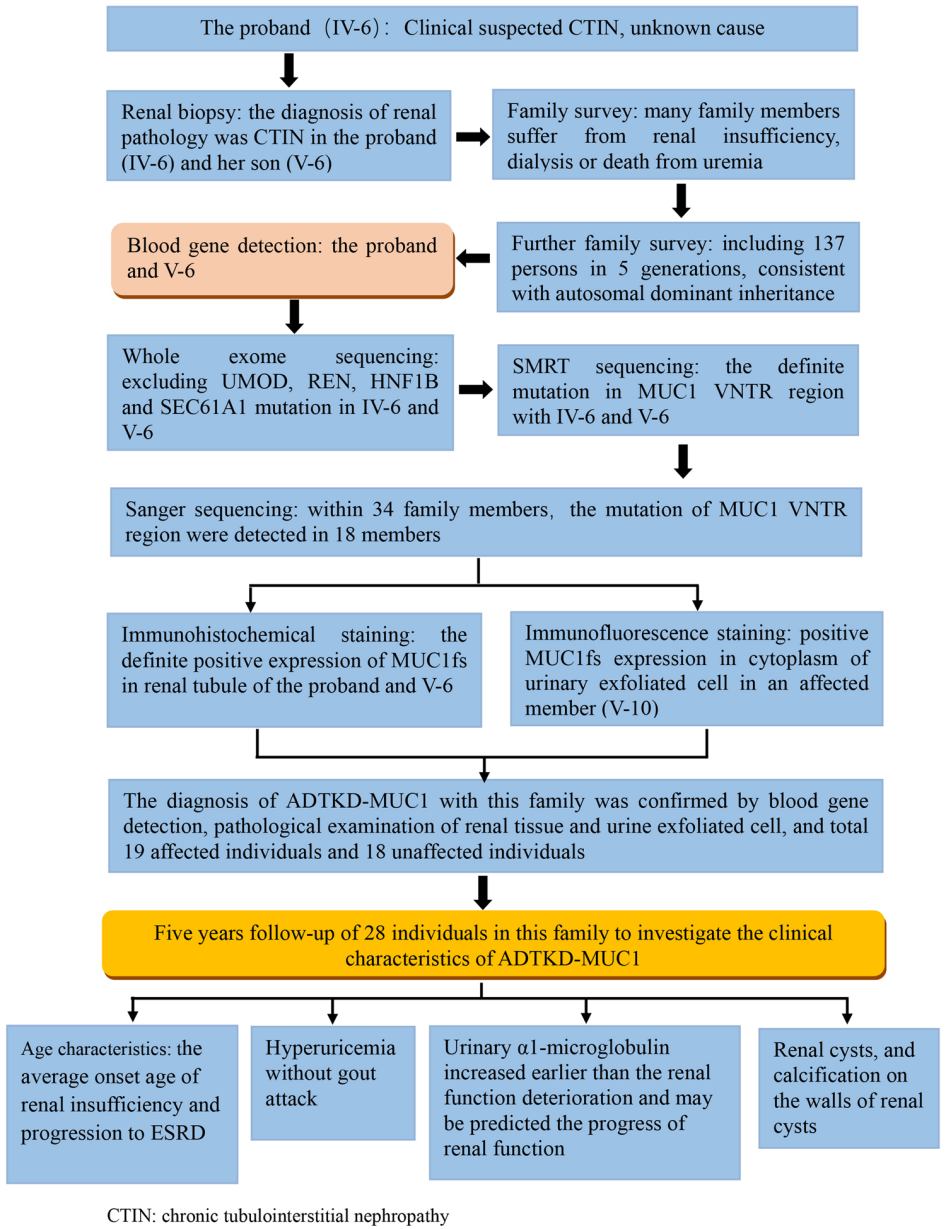
The flow chart of the diagnostic process for the ADTKD-MUC-1 family.

In summary, to the best of our knowledge, this is the first report on a large Chinese family with ADTKD-*MUC1*. A flow chart of the detailed process of the diagnosis and follow-up of this family is shown in Fig. 7. Because mutational analysis of *MUC1* is extremely difficult, we used SMRT sequencing to successfully detect *MUC1* mutations. However, this method is expensive and is not easy to apply widely for clinical diagnosis. We believe that MUC1fs immunostaining in kidney tissue and especially in urinary exfoliated cells could be helpful for early screening of individuals with suspected ADTKD-*MUC1*^14,18^. Interestingly, we observed significant differences in the levels of urinary α1-microglobulin between affected and unaffected family members. We also found that urinary α1-microglobulin levels increased earlier than SCr levels in affected individuals. Further studies should be performed to verify that urinary α1-microglobulin is a clinical marker for early indication of renal insufficiency and for prediction of the progression of renal dysfunction in individuals with ADTKD-*MUC1*.
